# A review of movement disorders in chemotherapy-induced neurotoxicity

**DOI:** 10.1186/s12984-021-00818-2

**Published:** 2021-01-25

**Authors:** Allison B. Wang, Stephen N. Housley, Ann Marie Flores, Sheetal M. Kircher, Eric J. Perreault, Timothy C. Cope

**Affiliations:** 1 Department of Biomedical Engineering, Northwestern University, Evanston, IL USA; 2 Department of Physical Therapy and Human Movement Sciences, Northwestern University, Chicago, IL USA; 3 Shirley Ryan AbilityLab, 355 E Erie St 21st Floor, Chicago, IL 60611 USA; 4 School of Biological Sciences, Georgia Institute of Technology, Atlanta, GA USA; 5 Department of Medical Social Sciences, Northwestern University, Chicago, IL USA; 6 Cancer Survivorship Institute, Robert H. Lurie Comprehensive Cancer Center of Northwestern University, Chicago, IL USA; 7 Department of Hematology and Oncology, Northwestern University, Chicago, IL USA; 8 Department of Physical Medicine and Rehabilitation, Northwestern University, Chicago, IL USA; 9 W.H. Coulter, Department of Biomedical Engineering, Emory University and Georgia Institute of Technology, Georgia Institute of Technology, Atlanta, GA USA; 10 Integrated Cancer Research Center, Parker H. Petit Institute for Bioengineering and Bioscience, Georgia Institute of Technology, Atlanta, GA USA

**Keywords:** Cancer, Chemotherapy, Neuropathy, CIPN, Sensorimotor dysfunction

## Abstract

Chemotherapy agents used in the standard treatments for many types of cancer are neurotoxic and can lead to lasting sensory and motor symptoms that compromise day-to-day movement functions in cancer survivors. To date, the details of movement disorders associated with chemotherapy are known largely through self-reported symptoms and functional limitations. There are few quantitative studies of specific movement deficits, limiting our understanding of dysfunction, as well as effective assessments and interventions. The aim of this narrative review is to consolidate the current understanding of sensorimotor disabilities based on quantitative measures in cancer survivors who received chemotherapy. We performed literature searches on PubMed and found 32 relevant movement studies. We categorized these studies into three themes based on the movement deficits investigated: (1) balance and postural control; (2) gait function; (3) upper limb function. This literature suggests that cancer survivors have increased postural sway, more conservative gait patterns, and suboptimal hand function compared to healthy individuals. More studies are needed that use objective measures of sensorimotor function to better characterize movement disabilities and investigate the underlying causes, as required for developing targeted assessments and interventions. By updating our understanding of movement impairments in this population, we identify significant gaps in knowledge that will help guide the direction of future research.

## Introduction

Chemotherapy agents used in the standard treatments for many types of cancer—including platinum compounds, taxanes, and vinca alkaloids—exhibit neurotoxic adverse effects. Depending on individual compounds, chemotherapy can damage the nervous system via various mechanisms (e.g., interference with axonal transport, mitochondrial damage, and altered ion channel activity) [1]. These adverse effects are commonly referred to as chemotherapy-induced peripheral neuropathy or neurotoxicity (CIPN). Although the ‘P’ in CIPN is included to describe damage to the peripheral nervous system, there is also evidence of central neurotoxicity [2, 3]. To acknowledge the central involvement that is not captured by peripheral neuropathy, we adopted CIN as chemotherapy-induced neurotoxicity for this review.

The prevalence of CIN varies from 19% to more than 85%, with the highest reported for platinum compounds (70–100%) and taxanes (11–87%) [4]. Although the mechanisms and prevalence of CIN may vary with drug type, the clinical presentations of patients with CIN share similar characteristics. Sensory symptoms associated with chemotherapy are most common and may include numbness/tingling, neuropathic pain, increased sensibility to hot/cold temperatures, and decreased vibration and pinprick sensitivity. Motor symptoms may include hyporeflexia, weakness, and muscle cramps. Autonomic symptoms, although less common, may include dizziness, hearing loss, and constipation [5, 6]. CIN symptoms can present immediately or progress after several cycles of treatment, and their severity usually increases with drug accumulation. These symptoms often improve over time after treatment cessation but can persist for years in a subset of patients, limiting their quality of life across the entire cancer illness trajectory [7–10]. A major issue associated with these sensory and motor symptoms is compromised movement function that contributes to functional impairments in day-to-day tasks [11, 12]. However, few studies quantify the specific movement deficits linked to sensory and motor signs and symptoms that reduce the quality of life in cancer survivors post-treatment. Specifying which components of a movement are impaired could focus the assessment of disability and recovery as well as possibly help identify more targeted interventions.

Descriptions of movement dysfunction associated with chemotherapy have come largely from self-reported symptoms and functional limitations, with few quantitative evaluations of movement function. Patient-reported outcome measures are the common clinical tools for assessing chemotherapy-induced neurotoxicity [13]. These measures are useful for tracking functional impairments and promoting communication of adverse symptoms and activity limitations among patients, oncologists, infusion nurses and personnel within cancer care teams [14]. However, self-reports are subjective, potentially biased (depending on the patient's recall) and inconsistently interpreted among patients and health care providers [15]. Most importantly, they provide no insight into the etiology of movement disability. Conventional neurological assessments, including nerve conduction studies, sensitivity of light touch, pin-prick and vibration, and deep-tendon reflexes may provide complementary information on CIN [13], though it is often noted that changes in neurophysiological signs do not reflect patient’s symptoms or function [16]. To address the limitations of self-reports and conventional neurological assessments on understanding the CIN-related movement dysfunction, quantitative and objective tools that directly evaluate the movement deficits are needed.

With the rising number of long-term survivors of cancer [17], there is a greater emphasis by the National Cancer Institute on improving quality of life and mitigating disability associated with the long-term effects of cancer treatment. A critical first step is to improve the understanding of chemotherapy-related movement deficits. Quantitative and instrumented movement studies have been widely used in other neurological populations to identify the characteristics and underlying causes of movement deficits [18–20]. In recent decades, more researchers have adopted this approach to investigate chemotherapy-induced movement dysfunction. Therefore, the objectives of this narrative review are to consolidate current knowledge of which movement functions are most commonly impaired in cancer survivors who received neurotoxic chemotherapy, to identify areas of research needed to improve the understanding of the movement deficits in this population, and to help guide improved assessment and treatments.

## Methods

**Fig. 1:**
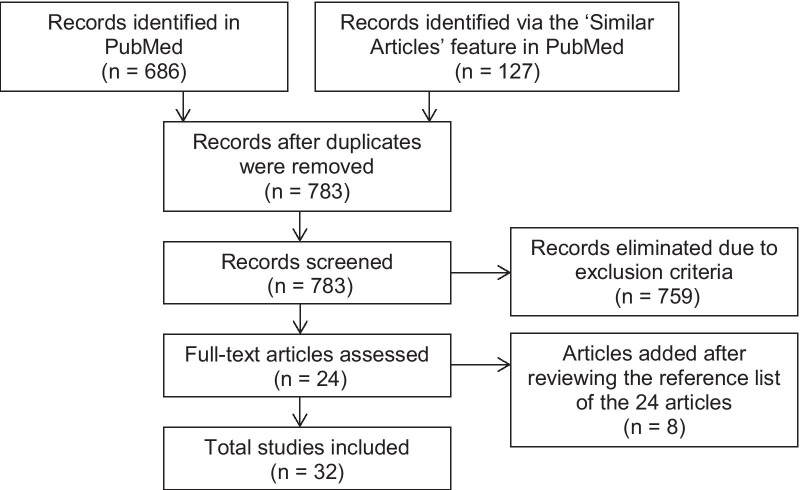
Flow diagram of article selection process

We performed a literature search on 5/15/2020 in PubMed, with a combination of search terms including derivations related to movement deficits (sensorimotor, movement, physical) and the disease (chemotherapy-induced neurotoxicity, chemotherapy-induced peripheral neuropathy, cancer, cancer patient, cancer survivor). Six hundred and eighty-six articles were identified from the search. Articles were included if they met all of the following inclusion criteria: (1) published within 2000–2020; (2) human subjects of any age, any cancer type; (3) most of the participants had received or were receiving neurotoxic chemotherapy, including platinum compounds, taxanes, and vinca alkaloids; (4) provided quantitative and instrumented assessments of movement deficits; (5) published in English. Articles were excluded if they (1) were a review or abstract; (2) assessed movement deficits only based on patient-reported outcome measures, functional outcome measures, or electrophysiological methods. Sixteen articles were selected after reviewing the titles and abstracts. We then used the ‘Similar Articles’ feature of Pubmed and identified 127 additional articles using Kneis et al. 2016 [21] as the search article. After reviewing the titles and abstracts of the 127 articles and checking for duplicates, we added eight articles to the list. We further reviewed the reference lists of the 24 selected articles and added eight additional articles. A total of 32 articles are included in this review (Fig. 1). The list of the 32 reviewed articles is shown in Tables 1, 2, and 3.

**Table 1:** Movement studies on balance and postural control

Authors	Population		Procedures	Examined variables	Results/Conclusions
	PAT	CT			
Muller 2020 [33]	Breast cancer patients: N = 35 (tested prior to and 3 weeks after neurotoxic treatment)	Gender, age, height, weight matched healthy controls: N = 35	Force plate measured CoP during bipedal stance EO, EC; semi-tandem stance EO, EC; monopedal stance EO	CoP: AP and ML mean velocity, 95% ellipse area, AP and ML mean frequency TNSr and TNSc NCS of the peroneal and sural nerves CIPN15-item questionnaire FES-I	PAT_post_ showed more sway than PAT_pre_ and CT. Occluding vision resulted in a greater sway increase in PAT_post_ than PAT_pre_ and CT. Sway of PAT_post_ correlated strongly with NCS, but weakly to none with the TNSr, TNSc, CIPN15 and FES-I.
Kneis 2020 [30]	Cancer patients with severe neuropathic symptoms: N = 8	Age, weight, height matched healthy controls: N = 15	Force plate measured CoP during bipedal stance EO, EC and during externally perturbed stance	CoP: AP and ML RMS, mean velocity, center frequency Angular excursion of lower and upper body segments NCS of the tibial and sural nerves Vibration sense Achilles and Patellar reflex FACT&GOG-Ntx	Sway amplitude and velocity were larger in PAT than CT. There was a significant group difference between PAT and CT that interacts with vision. PAT’s reactions to perturbations were smaller than CT’s.
Zahiri 2019 [29]	Cancer survivors: N = 82 (CIPN+: N = 58 and CIPN−: N = 24)	Age-matched controls: N = 57	IMU on the shins, thighs, and low back measured CoM sway during bipedal stance EO, EC	Area of ankle and hip sway, area of CoM sway, and ML CoM sway Vibration perception threshold FES-I	PAT had greater sway compared to CT with the largest effect observed in ankle sway during EC. The same trend held comparing CIPN+ to CIPN−. Vibration perception threshold was correlated with balance (ML CoM sway EO, area of CoM sway EC) and gait (stride time) parameters, and FES-I.
McCrary 2019 [40]	Cancer survivors 3 months to 5 years post neurotoxic therapy: N = 190 **(**symptomatic N = 129, asymptomatic N = 61)	NA	Swaymeter measured postural sway during bipedal stance EO, EC on ground, and bipedal stance EO, EC on foam	Total movement path length of CoM for all 4 tasks TNSc CIPN20 symptom index (first 4 items) CIPN Rasch-built Overall Disability Scale	Both symptomatic and asymptomatic patients had greater postural sway sum score compared to healthy elderly population. Patient-reported numbness/tingling, weakness, and balance deficits, age and vibration perception were strongly linked to the postural sway sum score.
Fino 2019 [27]	Female cancer survivors: N = 434 Classified into CIPN+ (N = 216) and CIPN− (N = 218)	Controls: N = 49	IMU at the lumbar spine measured tri-axial accelerations and angular velocities during bipedal stance EO	Principal component (PC) analysis of the IMU data FACT&GOG-Ntx Self-reported fall PC1: sway amplitude PC2: resultant and AP frequency and Jerk PC3: ML frequency	PAT had worse sway (PC1 and PC3) than CT. PAT fallers were likely to have smaller PC3 than PAT non-fallers PC3 was associated with falls if neuropathy was severe. PC2 was associated with falls when neuropathy is mild.
Morishita 2018 [26]	Cancer survivors: N = 19	Controls: N = 14	Force plate measured CoP during bipedal stance EO, EC	CoP: total sway length, sway area, the ratio between length and area TUG Grip strength Knee-extensor strength	PAT had increased sway area and decreased length/area during EO and decreased length/area during EC. PAT had decreased TUG score compared to control. TUG was correlated with muscle strength, but no sway parameters were related to muscle strength.
Schmitt 2017 [25]	Cancer patients (71% had chemo, 21% was actively receiving rad/chemo): N = 34	Age-matched (mean age) control: N = 34	Force plate measured CoP during bipedal stance on rigid and compliant surfaces with EO and EC	CoP: AP and ML RMS, AP and ML mean velocity, 95% ellipse area, AP and ML frequency, 95% power frequency of AP and ML sway	PAT had greater RMS and mean velocity in the ML direction and 95% sway area.
Monfort 2017 [32]	Patients with breast cancer prior to, during and 1–3 months after taxane chemotherapy: N = 33	NA	Force plate measured bipedal stance with EO A custom-built timing gate assessed gait speed and step length during fast forward 10-m walking	CoP: ML RMS sway mTNS CIPN20 C30 Brief Pain Inventory Gait: step length, walking speed	All measures progressively worsen over time. PAT_post_ had significant increased ML RMS sway. CIPN20 sensory subscale was significantly correlated with ML RMS.
Monfort 2016 [31]	Patients with breast cancer prior to, during and 1–3 months after taxane chemotherapy: N = 33	NA	Force plate measured bipedal stance with EO and EO	CoP: ML and AP RMS, ML and AP mean velocity, and 95% ellipse area	All parameters were impaired over the course of treatment; deficits were more pronounced during EC.
Kneis 2016 [21]	Patients with breast cancer and diagnosis of CIPN: N = 20	Sex, age, height, weight matched healthy controls: N = 16	Force plate measured CoP during bipedal and monopedal stance	Total CoP Ankle and hip angle H-reflex EMG of lower limb muscles to calculate co-contraction indices Neuropathy deficit score FACT&GOG-Ntx	CoP displacement was greater than CT during monopedal stance. Total CoP was correlated with co-contraction of soleus and tibialis anterior muscles and self-reported CIPN symptoms. PAT revealed prolonged H-wave latency, decreased H-reflex elicitability, and increased H-reflexed sensitivity from bi- to monopedal stance.
Monfort 2019 [64]	Cancer patients with mild CIPN: N = 8; Cancer patients with severe CIPN: N = 6	Cancer patients who had not received chemotherapy: N = 6	Balance pad assessed CoP during bipedal stance during seven conditions: (1) EO, head upright, rigid surface; (2) EC, head upright, rigid surface; (3) EO, head upright, foam surface; (4) EC, head upright, foam surface; (5) EO, head tilt, rigid surface; (6) EC, head tilt, rigid surface; (7) EC, head tilt, foam surface	CoP: 95% ellipse area, ML RMS, ML mean velocity and resultant mean velocity	CIPN+ group had significant deficits in summary CoP measures compared to that of CT and CIPN−. CIPN+ had greater ML sway deficits compared to CIPN−, particularly during rigid surface conditions.
Varedi 2018 [41]	Adult survivors of childhood acute lymphoblastic leukemia: N = 365	Sex-, race-, and age- (within 5 years) matched controls: N = 365	Dynamic posturography implemented the Sensory Organization Test (SOT)	SOT score, somatosensory ratio, vision ratio and vestibular ratio TUG 6MWT quality of life mTNS AROM of DF and PF Visual-motor processing speed	SOT score was not different between PAT and CT. Higher mTNS score was associated with longer TUG, shorter 6MWT, and reduced quality of life. Poorer visual-motor processing speed was associated with poorer SOT, TUG, and quality of life. PAT with impaired SOT score had lower vision and vestibular ratios than those without impaired SOT, but there was no difference in somatosensory ratio.
Ness 2013 [43]	Adult childhood cancer survivor at least 10 years post neurotoxic chemotherapy: N = 475	Healthy adults: N = 343	Sensory Organization Test: percent of time spent inside a 12-deg sway envelope during 6 conditions (SMART EquiTest): < 70% indicates problem with functional balance	mTNS peak dorsiflexion strength TUG 6MWT	12% PAT had problem with functional balance. 18% PAT had dorsiflexion weakness associated with Vincristine exposure. 20% PAT had sensory impairment associated with platinum exposure. Sensory impairment was associated with poor 6MWT and poor TUG.
Wampler 2007 [24]	Patients with breast cancer within 30 days of their final cycle of chemotherapy: N = 20	Age, weight and height matched healthy controls: N = 20	Sensory Organization Test: 6 standing conditions Force plate assessed CoP during bipedal stance with (1) EO head straight; (2) EC head tilt; (3) EC, head straight; (4) EO, head tilt	SOT score CoP: mean velocity Fullerton advanced balance scale TUG mTNS	PAT had worse performance on all measures compared to CT. The mTNS was moderately correlated with the total SOT score, explaining 44% of the variance in the SOT score.
Winters-Stone 2011 [46]	Breast cancer survivors within 2 years of treatment: N = 59	NA	Sensory Organization Test: 6 standing conditions	SOT score, equilibrium scores for each condition and sensory ratios Visual assessment battery Muscle mass Rep max leg press Timed stair climb Gait speed by 4 m walk Retrospective falls (last year) and prospective falls (6 months)	Past fallers had lower SOT scores with vestibular deficit patterns and took longer time to read letters on the contrast sensitivity chart. Vestibular score mediated fallers vs. non-fallers model.

*PAT* patients, *CT* controls, *CoP* center of pressure, *CoM* center of mass, *EO* eyes open, *EC* eyes closed, *AP* anteroposterior, *ML* mediolateral, *TNSr* total neuropathy score reduced version, *TNSc* total neuropathy score clinical version, *mTNS* modified total neuropathy score, *NCS* nerve conduction study, *CIPN* chemotherapy-induced peripheral neuropathy, *FES-I* Fall efficacy scale international version, *FACT&GOG-Ntx* Functional assessment of cancer therapy-gynecologic oncology group-neurotoxicity, *TUG* time-up-and-go test, *RMS* root mean square, *CIPN20* chemotherapy-induced peripheral neuropathy 20-item quality of life questionnaire, *C30* quality of life core questionnaire, *EMG* electromyography, *SOT* sensory organization test, *6MWT* six-minute walk test, *DF* dorsiflexion, *PF* plantarflexion, *AROM* active range of motion, *PROM* passive range of motion

**Table 2:** Movement studies on gait

Authors	Population		Procedures	Examined variables	Results/Conclusions
	PAT	CT			
Zahiri 2019 [29]	Cancer survivors: N = 82 (CIPN+ : N = 58 and CIPN−: N = 24)	Age-matched controls: N = 57	LEGSys assessed spatiotemporal gait parameters during 15-m walk using self-paced speed	Area of ankle and hip sway, area of CoM sway, and ML CoM sway Vibration perception threshold FES-I	PAT had greater sway compared to CT with the largest effect observed in ankle sway during EC. The same trend held comparing CIPN+ to CIPN−. Vibration perception threshold was correlated with balance (ML CoM sway EO, area of CoM sway EC) and gait (stride time) parameters, and FES-I.
Vallabha-josula 2019 [54]	Postmenopausal breast cancer survivors: N = 17	Age-matched controls: N = 17	Zeno walkway assessed self-paced and fast-paced forward walking, and self-paced backward walking on 16-ft walkaway	Speed, step length, step width, stance time, swing time, single support time, and double support time Strength: hand grip, chest press and leg press	PAT had 7% shorter step length, 8% slower gait speed compared to CT while walking both forward and backward. PAT had greater stance time variability during forward and fast forward conditions, but less during backward condition.
Hsieh 2019 [53]	CIPN+ : N = 9 CIPN−: N = 8	Age and sex-matched controls: N = 12	Zeno walkaway assessed spatiotemporal gait parameters during self-paced forward walking	FACT&GOG-Ntx Activities-specific Balance Confidence Scale Physiological Profile Assessment to assess overall fall risk	There were no group differences in gait speed, step length, or step width, but CIPN+ had greater step width variability and less step length variability than control.
Monfort 2019 [64]	Cancer patients within 6 weeks of completing chemotherapy CIPN− (mild to no symptoms): N = 9 CIPN+ (severe symptoms): N = 6	Cancer patient has not received chemotherapy: N = 6	An instrumented split belt treadmill assessed orbital gait stability during single-task and dual-task walking Bilateral lower extremity kinematics (motion capture)	Orbital stability Groton Max Learning Test to assess executive function CIPN20	CIPN+ group had worse executive function and was associated with decreased orbital stability during the dual-task condition. Chemotherapy maybe associated with impaired gait stability, but there was no evidence for an isolated association between CIPN and orbital stability.
Winters-Stone 2017 [49]	Women cancer survivors (average 6 years post treatment): N = 512 CIPN+ : N = 238 CIPN−: N = 274	NA	GAITRite system assessed spatiotemporal gait pattern during self-paced 4-m walk	Rep max leg press Short physical performance battery (5×STS, standing balance, 4-m walk speed) PRO: physical function and mobility disability; falls; severity of CIPN symptoms 1–4 Gait speed, step number, rate and length, stride length, base of support and % time in single support and double support	CIPN+ group took significantly more step, shorter step length, slower step time, shorter strides, and more time in double limb support compared to CIPN− group. Increased CIPN symptom severity was linearly associated with slower walking speed, slower chair stand time, and worse short physical performance battery score.
Monfort 2017 [32]	Patients with breast cancer prior to, during and 1–3 months after taxane chemotherapy: N = 33	NA	A custom-built timing gate assessed gait speed and step length during fast forward 10-m walking	CoP: ML RMS mTNS CIPN20 C30 Brief Pain Inventory Gait: step length, walking speed	All parameters progressively worsen over time; CIPN20 sensory subscale was significantly correlated with ML RMS. Gait speed and step length worsen during chemotherapy compared to baseline, but no difference was observed 1–3 months post chemotherapy.
Marshall 2017 [52]	Breast and colorectal cancer survivors with CIPN diagnosed by CTCAT: N = 8	Age- and morphologically-matched controls: N = 8	GAITRite system assessed spatiotemporal gait pattern during self-paced 8.2 m-walk	Gait: speed, step length, step time, swing time, single support time, base of support TUG	PAT had significant slower gait speed, shorter step length, and greater TUG score compared to CT.
Gilchrist 2016 [51]	Pediatric cancer patients with mTNS > 5 (average 6 months after treatment begins): N = 52	Age- and sex-matched healthy controls: N = 52	GAITRITE system measured spatiotemporal gait pattern during self-paced 14-ft walk (2 trials before and 2 trials after 6MWT-fatigue)	Gait speed, cadence, step length, base of support, time in single and double limb support Ped-mTNS Ankle ROM Strength and balance subscale of the Bruininks-Oseretsky Test of Motor Proficiency Ed 2	Before 6MWT, PAT had significantly slower speed and wider base of support; the decreased gait speed was related to decreased step length rather than cadence. After 6MWT, all parameters were significantly different between groups except double support time. PAT group had increased forefoot contact that reflects decreased eccentric control and fatigue of dorsiflexors. Decreased DF AROM and balance score explained the variance in step length the most for PAT group.
Beulertz 2016 [55]	Childhood cancer survivors who completed cancer treatment and < 5 years from diagnosis (85% received chemotherapy): N = 13	Age and gender-matched healthy controls: N = 13	Microgate Optogait 2D Gait Analysis System assessed spatiotemporal gait pattern during self-paced 2-m walk	Gait: step time, length and width, stride time and length, and gait cycle percentage DF AROM 6MWT to assess walking efficiency German Motor Test 6–18 to assess motor performance	DF AROM, gait (stance, swing and pre-sway phase) and walking efficiency were significantly impaired in PAT group compared to CT group. There was no group difference found in motor performance.
Wright 2017 [63]	Pediatric cancer patients who are receiving or have completed vincristine treatment and presented with CIPN: N = 17	Age-matched healthy controls: N = 10	3D motion capture and force plate analyzed kinematic and kinetic of gait during self-paced 8-m walk while surface EMG sensors recorded muscle activities on tibialis anterior and medial gastrocnemius muscles	Gait Deviation Index quantified the magnitude of gait deviation Kinematic, kinetic and spatiotemporal gait variables PROM DF Strength: DF and PF	Gait deviation was heterogenous in PAT group. PAT group had significantly less peak hip extension, knee flexion in loading, dorsiflexion at initial contact, plantarflexion at pre-swing, and dorsiflexion in swing, shorter step lengths, and lower ankle moments and powers compared to CT. PAT group also exhibited out of phase firing of MG and TA and high proportion of MG-TA co-activation compared to CT.

*PAT* patients, *CT* controls, *CoP* center of pressure, *CoM* center of mass, *AP* anteroposterior, *ML* mediolateral, *mTNS* modified total neuropathy score, *Ped-mTNS* pediatric version of the modified total neuropathy score, *CIPN* chemotherapy-induced peripheral neuropathy, *FES-I* Fall efficacy scale international version, *FACT&GOG-Ntx* Functional assessment of cancer therapy-gynecologic oncology group-neurotoxicity, *CIPN20* chemotherapy-induced peripheral neuropathy 20-item quality of life questionnaire, *C30* quality of life core questionnaire, *6MWT* six-minute walk test, *DF* dorsiflexion, *PF* plantarflexion, *AROM* active range of motion, *PROM* passive range of motion, *5* × *STS* five times sit to stand, *PRO* patient-reported outcome, *TA* tibialis anterior muscle, *MG* medial gastrocnemius muscle

**Table 3:** Movement studies on upper limb function

Authors	Population		Procedures	Examined variables	Results/Conclusions
	PAT	CT			
Osumi 2019 [69]	Cancer patients with perceived numbness from chemotherapy: N = 12	Age-matched controls: N = 12	Electromagnetic motion tracking system measured kinematic data while participants completed reach-to-grasp movement	Kinematic analysis: Reach and grasp movement (jerk index) Von Frey filament Numbness rating Motor function: # of grasp and release movements can be performed in 10 s	PAT group had impaired grasp jerk index compared to controls, but no significant difference was found in reach jerk index. Grasp jerk index was directly correlated with poor scores in sensory tests and hand grip-release test.
Reinders-Messelink 2001 [70]	Pediatric cancer patients receiving vincristine for acute lymphoblastic leukemia: N = 11	Age-matched healthy children: N = 11	A digitizer tablet and a force sensing pen measured quality of hand drawing during drawing tasks of different complexity	Quality of hand drawing: velocity, fluency, pause durations and pen pressure	PAT group drew slower, with longer pause durations and increased drawing pressure. PAT group were able to overcome the problems after vincristine was withdrawn, except for the increased drawing pressure.

*PAT* patients, *CT* controls

## Results and discussion

Thirty-two movement studies related to chemotherapy-induced neurotoxicity were identified. All of the reviewed movement studies focused on one of the three areas of movement function: (1) balance and postural control; (2) gait function; (3) upper limb function; therefore, we organized the results and discussion using these three themes.

### Characteristics of balance and postural control impairments in cancer survivors with CIN

Postural imbalance is one of the most common movement dysfunctions reported by cancer survivors. The ability to maintain postural balance, therefore, is an area commonly investigated in cancer survivors. Maintaining postural balance is a complex process involving various components of postural control, including a neural representation of body segments and position of the center of gravity, multisensory inputs that monitor the orientation and stability of body segments, and reactive or anticipatory responses for balance recovery after perturbations or postural stabilization during voluntary actions [22]. Most of the postural studies on cancer survivors evaluated this by measuring spontaneous postural sways (Table 1). Spontaneous postural sways are the natural oscillations of our body during normal stance, but they can become maladaptive in various pathologies [23]. Characterization of spontaneous postural sway in cancer survivors with CIN reveals postural instability. Nine studies compared the spontaneous sway of cancer survivors to that of healthy controls when standing with eyes open [21, 24–30]. Sway amplitude, including the root mean square (RMS) of the resultant sway [30], the mediolateral (ML) sway [25, 29], and the total sway area [25, 26, 28] were greater in cancer survivors than healthy controls. Sway velocity, including the mean velocity of ML sway [25, 28] and mean velocity of the resultant sway [24] were also greater in cancer survivors than that in controls. Three studies (the majority of participants had breast cancer) [31–33] assessed the longitudinal effect of chemotherapy on spontaneous sway and found that sway amplitude (area, ML RMS, AP RMS) and sway velocity (AP mean velocity, ML mean velocity) parameters worsen after treatment. This evidence suggests that cancer survivors with CIN are unstable in standing. Among the eight studies that investigated direction-dependent sway characteristics, seven identified ML sway deficits (RMS, velocity, and frequency) [25, 27–29, 31–33], whereas two also identified AP deficits [30, 33], suggesting that cancer survivors may be more unstable in frontal balance control. Impaired ML sway has been shown to be an important predictor of retrospective and prospective falls in older adults [34–36]. Fino et al. 2019 used principal component analyses on sway data and confirmed the association of ML sway frequency and falls in cancer survivors with severe neuropathic symptoms [27]. Unlike balance control in the sagittal plane that uses both distal ankle and proximal hip strategies, balance in the frontal plane is predominantly controlled via the load-unload mechanism accomplished by hip adductors and abductors while the ankle inversion-eversion plays a minimal role [37, 38]. The association of the impaired ML postural control with falls is likely due to the lack of a compensatory control scheme for ML balance.

These existing spontaneous sway studies suggest that there is excessive postural sway in cancer survivors, especially in the ML direction, but the factors underlying the amplified postural sway in this population have yet to be clarified. One hypothesis is that excessive postural sway is caused by the peripheral sensory neuropathy associated with CIN. This hypothesis is well motivated as the somatosensory system contributes more to postural stability than the visual and vestibular systems [39]. The peripheral sensory system constitutes different perceptual subsystems involving mechanoreceptors in skin, muscles, tendons, and ligaments, but the precise determination of the diminished peripheral sensory capability associated with CIN is not straightforward. Researchers investigating the relationship between increased postural sway and peripheral sensory neuropathy relied on various measures to assess the state of the peripheral sensory system, including subjective reports of sensory symptoms (e.g., severity of numbness/tingling, Functional Assessment of Cancer Therapy-Gynecologic Oncology Group-neurotoxicity (FACT&GOG-Ntx), and CIPN 20-item quality of life questionnaire (CIPN20)) [21, 29, 32, 33, 40], vibration perception threshold [29, 40], and conduction studies of peripheral nerves [33]. Composite scores that combine subjective symptoms and objective measures of sensory signs and reflexes (e.g., modified Total Neuropathy Score (mTNS)) [24, 41] were also used [33]. Zahiri et al. 2019 identified a significant correlation between the ML sway and plantar vibration perception threshold in patients reporting feet numbness/tingling [29]. Kneis et al. 2016 correlated the total center of pressure sway during monopedal stance with perceived symptom severity measured by the FACT&GOG-Ntx in breast cancer survivors with CIN [21]. Monfort et al. 2017 investigated the longitudinal effects of taxanes chemotherapy on breast cancer patients and found a significant correlation between ML sway and sensory symptoms measured by CIPN20 [32]. Muller et al. 2020 also investigated the longitudinal effect of neurotoxic chemotherapy, but on a cohort of patients with mixed cancer diagnoses. In contrast to Monfort et al. 2017, Muller et al. did not find a significant correlation between sway measures and sensory symptoms; instead, they found a significant correlation between sway measures and conduction speeds of the peroneal and sural nerves [33]. Wampler et al. 2007 and Varedi et al. 2018 observed a similar inconsistency. Both studies quantified a composite score of postural sway during six standing conditions and mTNS. Wampler et al. found a significant association between the composite score and the mTNS score in a group of breast cancer patients, but Varedi et al. studying a cohort of adult survivors of childhood acute lymphoblastic leukemia did not find the same association [24, 41]. Although different measures of postural sway and neuropathy were used in these correlational studies, the majority support an association of excessive postural sway with peripheral sensory deficits. The inconsistent findings between Monfort et al. 2017 and Muller et al. 2020 and between Wampler et al. 2007 and Varedi et al. 2018 suggest that the link between postural deficits and CIN might be specific to the type of cancer and/or type of chemotherapy used. Future studies should consider the impact of these variables on chemotherapy-induced impairments of posture and balance control.

The correlational studies, however, do not suffice to conclude a causal relationship between peripheral sensory deficits and excessive postural sway or rule out other contributing factors. In fact, McCrary et al. 2019 found that cancer patients, regardless of sensory symptoms, had greater postural sway compared to age-matched normative values. Among the five factors contributing to increased postural sway (patient-reported balance/mobility deficits, abnormal vibration, numbness/tingling, self-reported weakness, and age > 65), only two were related to peripheral sensory deficits [40]. These results suggest that motor deficits such as weakness may also affect postural balance [40, 42], but few have directly assessed their impact. One study with a cohort of mixed cancer types found no difference in grip or knee extension strength between control subjects and cancer survivors and no correlation between these strength measures and postural sway [26]. In contrast, a separate study on cancer survivors who had received vincristine chemotherapy found that impaired dorsiflexion strength was correlated with balance score [43]. These variable findings underscore the need for assessing the impact of motor function on postural control in more tightly controlled patient cohorts and treatment types, as it could be a major contributor to chemotherapy-induced disability along with sensory deficits.

Postural balance depends on the integration of sensory inputs from the somatosensory, visual, and vestibular systems to elicit appropriate motor responses [44]. Although current evidence suggests a link between CIN-induced somatosensory deficits and postural instability, it is not clear if there are also deficits in the visual and vestibular system contributing to postural instability and how cancer survivors adapt their control strategies. Systematically altering or removing one or more sensory inputs has been used to investigate the contribution of an individual sensory system to postural stability and the sensory integration process. Among the six studies that occluded vision to investigate the visual dependency of changes in postural sway, four observed a greater effect of visual occlusion on postural sway in cancer survivors than that in controls [28–31], whereas two did not [24, 26]. The greater weighting of the visual system by cancer survivors suggests potential deficits in the somatosensory and/or vestibular systems. Kneis et al. 2020 ruled out potential vestibular dysfunction via the rotational chair test [30]. They further dissociated the relative weighting of somatosensory and vestibular systems in postural control by perturbing standing posture using a tilting platform and measuring the subsequent excursions of the upper (shoulder-hip) and lower (hip-ankle) body and center of pressure displacements. They found that cancer survivors had smaller body excursions than controls in response to platform tilts, suggesting that cancer survivors use vestibular rather than proprioceptive cues for postural control as proprioceptive cues may drag the body along platform movements (greater body excursions), whereas vestibular cues would stabilize the body in space (smaller body excursions). A postural-control model fitting the experimental data was consistent with a down-weighting of the proprioceptive cues in cancer survivors. The underutilization of the somatosensory system was also supported qualitatively by Monfort et al. 2019 [28]. Their data revealed that the symptomatic group exhibited smaller postural deteriorations when somatosensory input was altered (standing on foam) compared to that of healthy controls and the asymptomatic group, implying that the symptomatic group relied less on somatosensory feedback for postural balance. Although it appears that cancer survivors rely more on the vestibular system for postural control, whether the vestibular function is intact after chemotherapy remains debatable. Kneis et al. 2020 is the only postural study that assessed vestibular function, finding no vestibular dysfunction in their cohort. However, the rate of abnormal vestibular function after chemotherapy ranges from 0 to 50% [45]. Furthermore, Wampler et al. 2007 found two of the largest postural sway differences between cancer survivors and controls occurred in standing conditions relying on vestibular input, suggestive of vestibular impairments [24]. This agrees with the study by Winters-Stone et al., which identified balance deficits of vestibular origin contributing to falls among breast cancer survivors who received chemotherapy, although the authors also assessed vision and identified an association of impaired visual contrast sensitivity with falls [46].

In summary, studies of sensory integration have revealed that cancer survivors underutilize somatosensory feedback for postural control, likely due to CIN-related somatosensory deficits. As a compensatory strategy, cancer survivors increase the weight of the visual and vestibular systems, but the summarized evidence indicates that this strategy compensates incompletely for the deficits in the somatosensory system during static standing. The extent to which the visual and vestibular dysfunction contribute to postural instability remains unclear, as few of the reviewed postural studies performed rigorous tests of these systems. Likewise, few studies performed detailed assessments of the motor system. Future studies should consider how the CIN-related motor function changes (i.e., muscle strength) affect postural stability. Kneis et al. 2020 presented a useful paradigm for investigating sensory integration strategies adapted by cancer survivors. However, the study was based on a small sample with severe balance deficits, so the conclusion cannot be extrapolated to cancer patients with different levels of CIN severity. The sample also consisted of mixed cancer types and treatments; whether there are cancer type-related, treatment-related differences, or interaction effects [47] remains to be investigated. Therefore, robust postural control studies with larger sample sizes and tightly controlled cancer and treatment types are needed to further clarify the postural control strategies adopted by cancer survivors.

### Characteristics of gait impairments in cancer survivors with CIN

Falls are common in cancer survivors. It is estimated that about 30% of cancer survivors fall every year [48], and individuals with CIN symptoms are 1.7–1.8 times more likely to fall than the asymptomatic individuals [7, 49]. The majority of falls occur during walking [50]; therefore, understanding walking behaviors in cancer survivors with CIN may provide information on how to prevent falls and fall-related injuries. Walking behavior is commonly characterized by the spatial and temporal parameters of gait, including step or stride length, step width, gait speed, single- or double-support, and swing time. Eight studies compared these gait parameters of cancer survivors with CIN to that of healthy controls (or asymptomatic patient group, or individuals prior to chemotherapy) and revealed that cancer survivors with CIN had impaired spatiotemporal gait pattern (Table 2) [29, 32, 49, 51–55]. During level ground walking with self-selected speed, six out of eight studies reported significantly decreased gait speed in the patient group [29, 32, 49, 51, 52, 54]. Other changes such as increased stride/step time [29, 53], decreased stride/step length [29, 32, 49, 51, 52, 54], increased double support time [29, 49, 55], and increased step width variability [53] were also reported. These gait changes reflect a conservative gait pattern [56], which is also observed in the population with diabetic neuropathy [19] and has been associated with fall risk in elderly populations [57, 58].

Similar to postural instability, this impaired gait pattern was shown to be associated with CIN-related neuropathy. Winters-Stone et al. 2017 found a significant association between lower walking speed and increasing numbness/tingling and discomfort in feet [49]. Zahiri et al. 2019 found a significant correlation between stride time and plantar vibration threshold [29]. Gilchrist et al. 2016 found a correlation of greater than 0.3 between step length and pediatric mTNS [51]. Although specific gait pattern changes like decreased step length and increased cadence can also be explained by decreased gait speed [59], increased gait variability appears to be related to deficits in somatosensory feedback. It has been suggested that sensory feedback is important for adjusting step-to-step limb trajectories and smoothing unexpected perturbation during locomotion [60, 61]. Deficits in sensory feedback, therefore, could have a greater influence on the variability of gait than the mean locomotor pattern. Wuehr et al. 2014 demonstrated that ML gait variability was highly sensitive to deficits in peripheral sensory feedback, irrespective of gait speed, supporting the important role of integrative sensory feedback for walking adjustment in this plane [59]. This hypothesis was consistent with the study of Hsieh et al. 2019, who found a greater step width variability in symptomatic cancer survivors than in healthy controls without a significant difference in gait speed, suggesting that locomotion instability observed in cancer survivors may be linked to deficits in sensory feedback [53].

Cancer survivors with CIN demonstrated conservative gait patterns characterized by slower gait speed, shorter step length, longer double support time, and greater ML gait variability. These altered gait patterns have been linked to somatosensory deficits associated with CIN [29, 49], but it remains unclear if other factors that contribute to stability during locomotion in healthy subjects also contribute to disability in cancer survivors. These include the visual and vestibular systems, spinal and supraspinal networks, and musculoskeletal functions [62]. For example, musculoskeletal impairments, such as impaired range of motion and decreased lower extremity strength, contribute to gait impairments in individuals with diabetic neuropathy, along with the well-documented sensory deficits in this population [19]. Currently, the prevalence of similar musculoskeletal impairments in cancer survivors remains unknown. Wright et al. 2017 used kinematic and kinetic analyses of gait in children with vincristine-induced neurotoxicity and speculated that the deviated gait pattern was related to decreased dorsiflexion range of motion, ankle weakness, and a high proportion of co-contraction in the medial gastrocnemius and tibialis anterior muscles [63]. Gilchrist et al. 2016 also found that decreased dorsiflexion range of motion and impaired balance score explained decreased step length the most [51]. These results are intriguing, but it is unclear if they are relevant to adult cancer survivors since both studies were performed on children. Co-contraction of medial gastrocnemius and tibialis anterior muscles has been documented as a safety strategy used by adult cancer survivors with CIN for balance control, but only in static standing tasks [21]. These results have been observed in pediatric cancer survivors during gait, and it will be useful to determine if a similar strategy is employed by adult cancer survivors. Monfort et al. 2019 is the only study that considered the role of cognition in gait stability [64]. They quantified gait stability in cancer survivors with CIN during single- and dual-task walking. They found that cancer survivors had similar gait stability during the single-task walking compared to healthy controls, but the stability cost was greater during the dual-task walking, and it was associated with poor executive function. The increased stability cost during dual-task walking could be due to the diminished sensory feedback associated with CIN that makes gait control more cognitively costly, but there was no evidence for an isolated association between CIN severity and gait stability. These results suggest that cognitive impairments in addition to CIN could contribute to gait impairments though more work is needed to evaluate the prevalence and relative importance of these contributions. Finally, we were unable to find any studies that evaluated the impact of chemotherapy on the visual and vestibular systems even though these are known to be central to unimpaired gait.

In summary, current evidence on the underlying causes of gait abnormality in cancer survivors remains limited. Musculoskeletal deficits at the ankles, including reduced range of motion and strength and increased muscular co-contraction, contribute to altered gait patterns in pediatric cancer survivors, but further kinematic and kinetic gait analyses are warranted to determine if similar musculoskeletal changes occur in adult cancer survivors. Future gait studies should also investigate how chemotherapy-related changes in the central nervous system (e.g., vision, vestibular, cognition) contribute to gait impairments.

### Characteristics of upper limb function impairments in cancer survivors with CIN

CIN-induced sensorimotor dysfunction not only contributes significantly to balance and gait dysfunction in cancer survivors but also plays a significant role in upper extremity dysfunction. Particularly, cancer survivors with CIN report difficulties with skilled hand function such as typing, writing, and buttoning a shirt [8, 65–68], but few studies have investigated the specific components of the impairments and contributions from the CIN-induced sensory or motor dysfunction (Table 3). Osumi et al. 2019 investigated one of the essential upper limb motor behaviors, reach-to-grasp movement, in cancer survivors with perceived numbness due to neurotoxic chemotherapy [69]. Their reach-to-grasp movement consisted of a reach component that primarily reflects the motor function of the proximal upper limb muscles and a thumb-index grasp component that requires fine control of hands and fingers. They found that cancer survivors had a significantly decreased smoothness during grasping but similar smoothness during reaching compared to healthy age-matched controls. The grasp smoothness was significantly correlated with hand sensory function, measured by tactile detection threshold and numbness rating, and hand motor function, measured by the hand grip-release test, suggesting that hand sensory and motor dysfunction may contribute to impaired thumb-index grasp smoothness. Reinders-Messelink et al. 2001 investigated handwriting dexterity in children undergoing vincristine chemotherapy for acute lymphoblastic leukemia [70] and found that pen pressure increased progressively during and six months after treatment, and the effect was most significant with the most complex drawing task. Other qualities of handwriting, such as velocity, dysfluency, pause duration, and accuracy, were not different between patients and healthy controls. It was speculated that increased pen pressure is a compensatory mechanism for vincristine-induced sensory impairments whereby increased pen pressure can, in turn, increase pen-paper friction, providing more kinesthetic information needed for handwriting tasks.

In summary, these two studies provide preliminary evidence of suboptimal hand function linked to CIN-related sensory and motor disturbances. However, since thumb-index grasp and handwriting only represent parts of skilled hand function, further studies are needed to investigate other skilled hand function and manual dexterity (e.g., power vs. precision grasp, prehensible vs. non-prehensible object manipulation) and how they are affected by CIN. Furthermore, neither study considered the compensatory effect of vision on task performance, thus potentially misidentifying the functional significance of CIN-induced sensory and motor dysfunction. Although incorporating vision is more functionally relevant and takes hand-eye-coordination into account, identifying the relative contribution of sensory and motor dysfunction independent of vision can be useful for identifying targets of intervention.

### Other factors to consider when investigating movement dysfunction in cancer survivors

Chemotherapy-induced neurotoxicity produces unique sensory and motor symptoms that contribute to dysfunction in postural control, gait, and upper limb function. While further research is warranted to fully characterize CIN movement dysfunction and its underlying causes, researchers should also consider other side effects of cancer and treatments, including fatigue, cognitive changes, and pain, when designing future studies. Cancer-related fatigue is common, with most studies reporting prevalence rates above 60% [71]. Cancer fatigue can have a peripheral component that is perceived as a sensation of weakness, which may be confounded with CIN-related motor symptoms [71]. It also can have a central component, defined as difficulty in initiating or maintaining voluntary physical and cognitive activities [2, 71], which could negatively affect attention and interfere with movement function, particularly during tasks that require greater cognitive loads (i.e., dual-task). Closely related to central fatigue is cognitive dysfunction in cancer survivors. It is estimated that 75% of patients might have measurable cognitive impairments during treatment, and 35% will continue to exhibit cognitive difficulty months to years following treatment [72]. These cognitive impairments range from changes in attention, memory, executive function, and psychomotor speed, related to the comorbid factors associated with cancer such as depression and anxiety and/or direct effects of chemotherapy/radiation and cancer itself [72]. Cognition and attention play important roles in the maintenance of balance and postural control [73]; therefore, these factors should be considered when interpreting the results of balance and postural impairments. Pain is another factor that can alter movement patterns [74]. Neuropathic pain related to chemotherapy, although not as common as numbness/tingling, can present in a substantial patient population [75]. Cancer patients may also present with pain originating from tumor excision, removal of a body part (i.e., breast), tumor-related spinal cord compression, bone metastasis, and radiation injuries depending on types of cancer and course of individual cancer treatment [76].

In summary, individuals with cancer might present with other side effects add to the CIN-induced sensory and motor symptoms. Side effects like fatigue, cognitive dysfunction, and pain could complicate the interpretation of movement dysfunction. Researchers should consider monitoring these side effects, if not controlling for them when investigating movement dysfunction linked to CIN.

## Conclusion

Motivated by improving the management of chemotherapy-related movement dysfunction, this literature review evaluated 32 studies and consolidated the knowledge of common movement disabilities in cancer survivors who received chemotherapy. Overall, cancer survivors with chemotherapy-induced neurotoxicity have been shown to present with increased postural sway, conservative gait patterns, and suboptimal hand function, but the current understanding of CIN-related movement function changes is far from comprehensive.

We identified a number of areas where more information is needed. Cancer survivors with CIN report a wide range of dysfunction in gross mobility (e.g., balance, walking, climbing stairs, and driving) and fine motor skills (e.g., tying shoes, buttoning clothes, writing, typing, opening lids, and cooking) [7, 8, 12, 65–68, 77]. The majority of the reviewed studies focused on quantifying postural and gait impairments, which are useful for understanding balance and walking dysfunction. However, the understanding of other mobility limitations, such as driving and stair climbing, is still lacking. Furthermore, current evidence is not clear on the underlying causes of gait and postural dysfunction. CIN-related somatosensory deficits likely play a role, but more research is needed to control and test other factors, including motor and central factors, to delineate their relative contributions to gait and postural dysfunction. Similarly, the two studies on the upper extremity have identified some important deficits of hand function, including impaired smoothness in grasping and increased pen pressure in writing, but more studies are needed to understand other aspects of fine motor skills and manual dexterity.

There are currently no effective treatments for CIN. Many early reports suggest a possible beneficial effect of exercise (see reviews [11, 78, 79]). However, most exercise studies took a multimodal approach. We do not know what the best therapies are, nor do we have objective measures to determine if the therapies that we are using are effective in treating CIN, or they simply lead to compensation. Knowledge gaps in the objective characterization and underlying causes of CIN-related movement dysfunction present formidable barriers. To begin to address these outstanding issues, researchers and clinicians should work in concert to integrate and act upon objective measures deployed across the cancer treatment continuum. While this review characterized significant heterogeneity in evaluative tools and methodology for understanding CIN-related movement dysfunction, Kneis et al. provide a framework on which to build future clinical studies [30]. By integrating more sensitive and reliable tools, the authors not only gained information about baseline group level deficits resulting from the effects of chemotherapy but also the capability to precisely monitor treatment effects. Both advantages outlined will be crucial for discovering factors associated with sensorimotor deficits and making rigorous determinations on the efficacy of proposed treatments.

In summary, we identify significant knowledge gaps in CIN-related movement dysfunction and recommend frameworks for future clinical studies. Filling these gaps will help improve the clinical understanding of CIN-related movement dysfunction and guide the development of targeted assessments and treatments.

## Data Availability

Not applicable.

## Abbreviations

CIPN: Chemotherapy-induced peripheral neuropathy or neurotoxicity
CIN: Chemotherapy-induced neurotoxicity
PAT: Patients
CT: Controls
CoP: Center of pressure
CoM: Center of mass
EO: Eyes open
EC: Eyes closed
AP: Anteroposterior
ML: Mediolateral
TNSr: Total neuropathy score reduced version
TNSc: Total neuropathy score clinical version
mTNS: Modified total neuropathy score
NCS: Nerve conduction study
FES-I: Fall efficacy scale international version
FACT&GOG-Ntx: Functional assessment of cancer therapy-gynecologic oncology group-neurotoxicity
TUG: Time-up-and-go test
RMS: Root mean square
CIPN20: Chemotherapy-induced peripheral neuropathy 20-item quality of life questionnaire
C30: Quality of life core questionnaire
EMG: Electromyography
SOT: Sensory organization test
6MWT: Six-minute walk test
DF: Dorsiflexion
AROM: Active range of motion
PROM: Passive range of motion
TA: Tibialis anterior muscle
MG: Medial gastrocnemius muscle

## References

[1] Zajaczkowska R, Kocot-Kepska M, Leppert W, Wrzosek A, Mika J, Wordliczek J (2019). Mechanisms of chemotherapy-induced peripheral neuropathy. Int J Mol Sci.

[2] Housley SN, Nardelli P, Powers RK, Rich MM, Cope TC (2020). Chronic defects in intraspinal mechanisms of spike encoding by spinal motoneurons following chemotherapy. Exp Neurol.

[3] Sioka C, Kyritsis AP (2009). Central and peripheral nervous system toxicity of common chemotherapeutic agents. Cancer Chemother Pharmacol.

[4] Banach M, Juranek JK, Zygulska AL (2017). Chemotherapy-induced neuropathies—a growing problem for patients and health care providers. Brain Behav.

[5] Argyriou AA, Bruna J, Marmiroli P, Cavaletti G (2012). Chemotherapy-induced peripheral neurotoxicity (CIPN): an update. Crit Rev Oncol Hematol.

[6] Kerckhove N, Collin A, Conde S, Chaleteix C, Pezet D, Balayssac D (2017). Long-term effects, pathophysiological mechanisms, and risk factors of chemotherapy-induced peripheral neuropathies: a comprehensive literature review. Front Pharmacol.

[7] Bao T, Basal C, Seluzicki C, Li SQ, Seidman AD, Mao JJ (2016). Long-term chemotherapy-induced peripheral neuropathy among breast cancer survivors: prevalence, risk factors, and fall risk. Breast Cancer Res Treat.

[8] Mols F, Beijers T, Lemmens V, van den Hurk CJ, Vreugdenhil G, van de Poll-Franse LV (2013). Chemotherapy-induced neuropathy and its association with quality of life among 2- to 11-year colorectal cancer survivors: results from the population-based PROFILES registry. J Clin Oncol.

[9] Shah A, Hoffman EM, Mauermann ML, Loprinzi CL, Windebank AJ, Klein CJ, Staff NP (2018). Incidence and disease burden of chemotherapy-induced peripheral neuropathy in a population-based cohort. J Neurol Neurosurg Psychiatry.

[10] Tofthagen C, Overcash J, Kip K (2012). Falls in persons with chemotherapy-induced peripheral neuropathy. Support Care Cancer.

[11] Knoerl R, Gilchrist L, Kanzawa-Lee GA, Donohoe C, Bridges C, Smith EML (2020). Proactive rehabilitation for chemotherapy-induced peripheral neuropathy. Semin Oncol Nurs.

[12] Komatsu H, Yagasaki K, Komatsu Y, Yamauchi H, Yamauchi T, Shimokawa T, Doorenbos AZ (2019). Falls and functional impairments in breast cancer patients with chemotherapy-induced peripheral neuropathy. Asia Pac J Oncol Nurs.

[13] Park SB, Alberti P, Kolb NA, Gewandter JS, Schenone A, Argyriou AA (2019). Overview and critical revision of clinical assessment tools in chemotherapy-induced peripheral neurotoxicity. J Peripher Nerv Syst.

[14] Black N (2013). Patient reported outcome measures could help transform healthcare. BMJ.

[15] Knoerl R, Smith EML, Han A, Doe A, Scott K, Berry DL (2019). Characterizing patient-clinician chemotherapy-induced peripheral neuropathy assessment and management communication approaches. Patient Educ Couns.

[16] McCrary JM, Goldstein D, Sandler CX, Barry BK, Marthick M, Timmins HC, Li T, Horvath L, Grimison P, Park SB (2019). Exercise-based rehabilitation for cancer survivors with chemotherapy-induced peripheral neuropathy. Support Care Cancer.

[17] Siegel RL, Miller KD, Jemal A (2019). Cancer statistics, 2019. CA Cancer J Clin.

[18] Das S, Trutoiu L, Murai A, Alcindor D, Oh M, De la Torre F, Hodgins J (2011). Quantitative measurement of motor symptoms in Parkinson's disease: a study with full-body motion capture data. IEEE Eng Med Biol Soc.

[19] Mustapa A, Justine M, Mustafah NM, Jamil N, Manaf H (2016). Postural control and gait performance in the diabetic peripheral neuropathy: a systematic review. Biomed Res Int.

[20] Schwarz A, Kanzler CM, Lambercy O, Luft AR, Veerbeek JM (2019). Systematic review on kinematic assessments of upper limb movements after stroke. Stroke.

[21] Kneis S, Wehrle A, Freyler K, Lehmann K, Rudolphi B, Hildenbrand B, Bartsch HH, Bertz H, Gollhofer A, Ritzmann R (2016). Balance impairments and neuromuscular changes in breast cancer patients with chemotherapy-induced peripheral neuropathy. Clin Neurophysiol.

[22] Massion J (1994). Postural control system. Curr Opin Neurobiol.

[23] Maurer C, Peterka RJ (2005). A new interpretation of spontaneous sway measures based on a simple model of human postural control. J Neurophysiol.

[24] Wampler MA, Topp KS, Miaskowski C, Byl NN, Rugo HS, Hamel K (2007). Quantitative and clinical description of postural instability in women with breast cancer treated with taxane chemotherapy. Arch Phys Med Rehabil.

[25] Schmitt AC, Repka CP, Heise GD, Challis JH, Smith JD (2017). Comparison of posture and balance in cancer survivors and age-matched controls. Clin Biomech.

[26] Morishita S, Mitobe Y, Tsubaki A, Aoki O, Fu JB, Onishi H, Tsuji T (2018). Differences in balance function between cancer survivors and healthy subjects: a pilot study. Integr Cancer Ther.

[27] Fino PC, Horak FB, El-Gohary M, Guidarelli C, Medysky ME, Nagle SJ, Winters-Stone KM (2019). Postural sway, falls, and self-reported neuropathy in aging female cancer survivors. Gait Posture.

[28] Monfort SM, Pan X, Loprinzi CL, Lustberg MB, Chaudhari AMW (2019). Impaired postural control and altered sensory organization during quiet stance following neurotoxic chemotherapy: a preliminary study. Integr Cancer Ther.

[29] Zahiri M, Chen KM, Zhou H, Nguyen H, Workeneh BT, Yellapragada SV, Sada YH, Schwenk M, Najafi B (2019). Using wearables to screen motor performance deterioration because of cancer and chemotherapy-induced peripheral neuropathy (CIPN) in adults—toward an early diagnosis of CIPN. J Geriatr Oncol.

[30] Kneis S, Wehrle A, Dalin D, Wiesmeier IK, Lambeck J, Gollhofer A, Bertz H, Maurer C (2020). A new approach to characterize postural deficits in chemotherapy-induced peripheral neuropathy and to analyze postural adaptions after an exercise intervention. BMC Neurol.

[31] Monfort SM, Pan X, Patrick R, Singaravelu J, Loprinzi CL, Lustberg MB, Chaudhari AMW (2016). Natural history of postural instability in breast cancer patients treated with taxane-based chemotherapy: a pilot study. Gait Posture.

[32] Monfort SM, Pan X, Patrick R, Ramaswamy B, Wesolowski R, Naughton MJ, Loprinzi CL, Chaudhari AMW, Lustberg MB (2017). Gait, balance, and patient-reported outcomes during taxane-based chemotherapy in early-stage breast cancer patients. Breast Cancer Res Treat.

[33] Muller J, Ringhof S, Vollmer M, Jager LB, Stein T, Weiler M, Wiskemann J (2020). Out of balance—postural control in cancer patients before and after neurotoxic chemotherapy. Gait Posture.

[34] Maki BE, Holliday PJ, Topper AK (1994). A prospective-study of postural balance and risk of falling in an ambulatory and independent elderly population. J Gerontol.

[35] Lord SR, Rogers MW, Howland A, Fitzpatrick R (1999). Lateral stability, sensorimotor function and falls in older people. J Am Geriatr Soc.

[36] Melzer I, Benjuya N, Kaplanski J (2004). Postural stability in the elderly: a comparison between fallers and non-fallers. Age Ageing.

[37] Bonnet CT, Lepeut M (2011). Proximal postural control mechanisms may be exaggeratedly adopted by individuals with peripheral deficiencies: a review. J Motor Behav.

[38] Termoz N, Halliday SE, Winter DA, Frank JS, Patla AE, Prince F (2008). The control of upright stance in young, elderly and persons with Parkinson's disease. Gait Posture.

[39] Simoneau GG, Ulbrecht JS, Derr JA, Cavanagh PR (1995). Role of somatosensory input in the control of human posture. Gait Posture.

[40] McCrary JM, Goldstein D, Trinh T, Timmins HC, Li T, Menant J, Friedlander M, Lewis CR, Hertzberg M, O'Neill S (2019). Balance deficits and functional disability in cancer survivors exposed to neurotoxic cancer treatments. J Natl Compr Canc Netw.

[41] Varedi M, Lu L, Howell CR, Partin RE, Hudson MM, Pui CH, Krull KR, Robison LL, Ness KK, McKenna RF (2018). Peripheral neuropathy, sensory processing, and balance in survivors of acute lymphoblastic leukemia. J Clin Oncol.

[42] Gewandter JS, Fan L, Magnuson A, Mustian K, Peppone L, Heckler C, Hopkins J, Tejani M, Morrow GR, Mohile SG (2013). Falls and functional impairments in cancer survivors with chemotherapy-induced peripheral neuropathy (CIPN): a University of Rochester CCOP study. Support Care Cancer.

[43] Ness KK, Jones KE, Smith WA, Spunt SL, Wilson CL, Armstrong GT, Srivastava DK, Robison LL, Hudson MM, Gurney JG (2013). Chemotherapy-related neuropathic symptoms and functional impairment in adult survivors of extracranial solid tumors of childhood: results from the St. Jude Lifetime Cohort Study. Arch Phys Med Rehabil.

[44] Jáuregui-Renaud K, Souayah N (2013). Postural balance and peripheral neuropathy. Peripheral neuropathy—a new insight into the mechanism, evaluation and management of a complex disorder.

[45] Prayuenyong P, Taylor JA, Pearson SE, Gomez R, Patel PM, Hall DA, Kasbekar AV, Baguley DM (2018). Vestibulotoxicity associated with platinum-based chemotherapy in survivors of cancer: a scoping review. Front Oncol.

[46] Winters-Stone KM, Torgrimson B, Horak F, Eisner A, Nail L, Leo MC, Chui S, Luoh SW (2011). Identifying factors associated with falls in postmenopausal breast cancer survivors: a multi-disciplinary approach. Arch Phys Med Rehabil.

[47] Housley SN, Nardelli P, Carrasco D, Rotterman TM, Pfahl E, Matyunina LV, McDonald JF, Cope TC (2020). Cancer exacerbates chemotherapy-induced sensory neuropathy. Cancer Res.

[48] Stone CA, Lawlor PG, Kenny RA (2011). How to identify patients with cancer at risk of falling: a review of the evidence. J Palliat Med.

[49] Winters-Stone KM, Horak F, Jacobs PG, Trubowitz P, Dieckmann NF, Stoyles S, Faithfull S (2017). Falls, functioning, and disability among women with persistent symptoms of chemotherapy-induced peripheral neuropathy. J Clin Oncol.

[50] Reimann H, Fettrow T, Thompson ED, Jeka JJ (2018). Neural control of balance during walking. Front Physiol.

[51] Gilchrist L, Tanner L (2016). Gait patterns in children with cancer and vincristine neuropathy. Pediatr Phys Ther.

[52] Marshall TF, Zipp GP, Battaglia F, Moss R, Bryan S (2017). Chemotherapy-induced-peripheral neuropathy, gait and fall risk in older adults following cancer treatment. J Cancer Res Pract.

[53] Hsieh KL, Trinh L, Sosnoff JJ (2019). Gait variability is altered in cancer survivors with self-reported neuropathy. Gait Posture.

[54] Vallabhajosula S, Deaterly CD, Madzima TA (2019). Comparison of forward and backward gait characteristics between those with and without a history of breast cancer. Gait Posture.

[55] Beulertz J, Bloch W, Prokop A, Rustler V, Fitzen C, Herich L, Streckmann F, Baumann FT (2016). Limitations in ankle dorsiflexion range of motion, gait, and walking efficiency in childhood cancer survivors. Cancer Nurs.

[56] Menz HB, Lord SR, Fitzpatrick RC (2003). Age-related differences in walking stability. Age Ageing.

[57] Pamoukdjian F, Paillaud E, Zelek L, Laurent M, Levy V, Landre T, Sebbane G (2015). Measurement of gait speed in older adults to identify complications associated with frailty: a systematic review. J Geriatr Oncol.

[58] Thaler-Kall K, Peters A, Thorand B, Grill E, Autenrieth CS, Horsch A, Meisinger C (2015). Description of spatio-temporal gait parameters in elderly people and their association with history of falls: results of the population-based cross-sectional KORA-Age study. BMC Geriatr.

[59] Wuehr M, Schniepp R, Schlick C, Huth S, Pradhan C, Dieterich M, Brandt T, Jahn K (2014). Sensory loss and walking speed related factors for gait alterations in patients with peripheral neuropathy. Gait Posture.

[60] Nashner LM (1980). Balance adjustments of humans perturbed while walking. J Neurophysiol.

[61] Gandevia SC, Burke D, Cordo P, Harnad S (1994). Does the nervous system depend on kinesthetic information to control natural limb movements?. Movement control.

[62] Takakusaki K (2017). Functional neuroanatomy for posture and gait control. J Mov Disord.

[63] Wright MJ, Twose DM, Gorter JW (2017). Gait characteristics of children and youth with chemotherapy induced peripheral neuropathy following treatment for acute lymphoblastic leukemia. Gait Posture.

[64] Monfort SM, Pan X, Loprinzi CL, Lustberg MB, Chaudhari AMW (2019). Exploring the roles of central and peripheral nervous system function in gait stability: preliminary insights from cancer survivors. Gait Posture.

[65] Bennett BK, Park SB, Lin CS, Friedlander ML, Kiernan MC, Goldstein D (2012). Impact of oxaliplatin-induced neuropathy: a patient perspective. Support Care Cancer.

[66] Driessen CM, de Kleine-Bolt KM, Vingerhoets AJ, Mols F, Vreugdenhil G (2012). Assessing the impact of chemotherapy-induced peripheral neurotoxicity on the quality of life of cancer patients: the introduction of a new measure. Support Care Cancer.

[67] Speck RM, DeMichele A, Farrar JT, Hennessy S, Mao JJ, Stineman MG, Barg FK (2012). Scope of symptoms and self-management strategies for chemotherapy-induced peripheral neuropathy in breast cancer patients. Support Care Cancer.

[68] Wang M, Cheng HL, Lopez V, Sundar R, Yorke J, Molassiotis A (2019). Redefining chemotherapy-induced peripheral neuropathy through symptom cluster analysis and patient-reported outcome data over time. BMC Cancer.

[69] Osumi M, Sumitani M, Abe H, Otake Y, Kumagaya SI, Morioka S (2019). Kinematic evaluation for impairment of skilled hand function in chemotherapy-induced peripheral neuropathy. J Hand Ther.

[70] Reinders-Messelink HA, Schoemaker MM, Snijders TA, Göeken LN, Bökkerink JP, Kamps WA (2001). Analysis of handwriting of children during treatment for acute lymphoblastic leukemia. Med Pediatr Oncol.

[71] Ryan JL, Carroll JK, Ryan EP, Mustian KM, Fiscella K, Morrow GR (2007). Mechanisms of cancer-related fatigue. Oncologist.

[72] Pendergrass JC, Targum SD, Harrison JE (2018). Cognitive impairment associated with cancer: a brief review. Innov Clin Neurosci.

[73] Paul L, Ellis BM, Leese GP, McFadyen AK, McMurray B (2009). The effect of a cognitive or motor task on gait parameters of diabetic patients, with and without neuropathy. Diabet Med.

[74] Hodges PW, Tucker K (2011). Moving differently in pain: a new theory to explain the adaptation to pain. Pain.

[75] Wolf SL, Barton DL, Qin R, Wos EJ, Sloan JA, Liu H, Aaronson NK, Satele DV, Mattar BI, Green NB (2012). The relationship between numbness, tingling, and shooting/burning pain in patients with chemotherapy-induced peripheral neuropathy (CIPN) as measured by the EORTC QLQ-CIPN20 instrument, N06CA. Support Care Cancer.

[76] Swarm RA, Paice JA, Anghelescu DL, Are M, Bruce JY, Buga S, Chwistek M, Cleeland C, Craig D, Gafford E (2019). Adult cancer pain, version 3.2019, NCCN Clinical Practice Guidelines in Oncology. J Natl Compr Canc Netw.

[77] Padman S, Lee J, Kumar R, Slee M, Hakendorf P, Richards A, Koczwara B, Kichenadasse G, Sukumaran S, Roy A (2015). Late effects of oxaliplatin-induced peripheral neuropathy (LEON)–cross-sectional cohort study of patients with colorectal cancer surviving at least 2 years. Support Care Cancer.

[78] Duregon F, Vendramin B, Bullo V, Gobbo S, Cugusi L, Di Blasio A, Neunhaeuserer D, Zaccaria M, Bergamin M, Ermolao A (2018). Effects of exercise on cancer patients suffering chemotherapy-induced peripheral neuropathy undergoing treatment: a systematic review. Crit Rev Oncol Hematol.

[79] Kanzawa-Lee GA, Larson JL, Resnicow K, Smith EML (2020). Exercise effects on chemotherapy-induced peripheral neuropathy: a comprehensive integrative review. Cancer Nurs.

